# Heat strain and mortality effects of prolonged central European heat wave—an example of June 2019 in Poland

**DOI:** 10.1007/s00484-021-02202-0

**Published:** 2021-10-26

**Authors:** Krzysztof Błażejczyk, Robert Twardosz, Piotr Wałach, Kaja Czarnecka, Anna Błażejczyk

**Affiliations:** 1 Climate Impacts Laboratory, Institute of Geography and Spatial Organization, Polish Academy of Sciences, Twarda 51/55, 00-818 Warszawa, Poland; 2 Faculty of Geography and Geology, Jagiellonian University in Cracow, Gronostajowa 7, 30-387 Kraków, Poland; 3 Institute of Meteorology and Water Management, National Research Institute, Borowego 14, 30-215 Kraków, Poland; 4 Laboratory of Bioclimatology and Environmental Ergonomic, Łukowska 17/55, 04-133 Warszawa, Poland

**Keywords:** Heat strain, Extreme heat wave, Heat-related mortality, UTCI

## Abstract

The occurrence of long-lasting severe heat stress, such as in July–August 2003, July 2010, or in April–May 2018 has been one of the biggest meteorological threats in Europe in recent years. The paper focuses on the biometeorological and mortality effects of the hot June that was observed in Central Europe in 2019. The basis of the study was hourly and daily Universal Thermal Climate Index (UTCI) values at meteorological stations in Poland for June 2019. The average monthly air temperature and UTCI values from 1951 to 2018 were analysed as background. Grosswetterlagen calendar of atmospheric circulation was used to assess synoptic conditions of heat wave. Several heat strain measures were applied : net heat storage (S), modelled heart rate (HR), sultriness (HSI), and UTCI index. Actual total mortality (TM) and modelled strong heat-related mortality (SHRM) were taken as indicators of biometeorological consequences of the hot June in 2019. The results indicate that prolonged persistence of unusually warm weather in June 2019 was determined by the synoptic conditions occurring over the European region and causing advection of tropical air. They led to the emergence of heat waves causing 10% increase in TM and 5 times bigger SHRM then in preceding 10 years. Such increase in SHRM was an effect of overheating and overload of circulatory system of human organism.

**Supplementary Information:**

The online version contains supplementary material available at 10.1007/s00484-021-02202-0.

## Introduction

Poland is located entirely in the temperate climate zone of Central Europe, which means a moderate inflow of solar radiation and the dominance of western circulation. In this part of Europe, frequent movement of lows with the accompanying atmospheric fronts and advection of air masses with different, sometimes even contrasting, thermal properties are the cause of the high variability of weather conditions from day to day (Błażejczyk, 2006). Depending on the incoming air masses, extreme hot or cold conditions may occur. Sometimes such extreme conditions can persist for a long time, which is usually caused by the development of high pressure blocking systems over eastern Europe (Twardosz and Batko, 2012; Schneidereit et al., 2012; Tishchenko et al., 2013).

There are well-recognised impacts of high air temperatures on the human organism and human activity. For example, Cheshire (2016), Błażejczyk et al. (2018), Gasparrini et al. (2015), Kuchcik (2017, 2021), di Napoli et al. (2018), Nastos and Matzarakis (2012), Revich and Shaposhnikov (2012) as well as Urban and Kysely (2014) reported an increase in mortality and morbidity in hot conditions. Kjellstrom and Lemke (2012), Bröde et al. (2013) and Gao et al. (2018) observed a reduction of work effectiveness in heat. Many authors (e.g. Miszuk 2021; Błażejczyk and Kunert, 2011; Owczarek et al., 2019) assessed the impacts of heat stress on bioclimatic potential for tourism and recreation. The research identified heat stress as a factor limiting possibility of intensive forms of recreational activity.

At the beginning of the twenty-first century, extremely hot months and entire seasons began to occur in various parts of Europe (Twardosz and Kossowska-Cezak, 2015; Twardosz et al., 2021). In the first decade, an extremely hot summer occurred in 2003 inwestern Europe and in 2010 ineastern part of the continent (Twardosz and Kossowska-Cezak, 2015, 2019, 2021). In the second decade, an unusually hot summer occurred in 2015 (IMGW-PIB 2015a, b; Hoy et al., 2016; Wypych et al., 2017; Twardosz and Kossowska-Cezak, 2021; Krzyżewska and Dyer, 2018), as well as April and May in 2018, which mainly covered vast areas of Central Europe, with the greatest heat intensity in Poland (Twardosz, 2019). In August 2015, the largest temperature anomaly of 5.9 °C(with standard deviation of 4.4) occurred in south-western Poland (Twardosz and Kossowska-Cezak, 2021). Even larger anomalies (6 °C) appeared in April 2018 insouth-eastern Poland (Twardosz, 2019). A year after the anomalously warm months of 2018, June 2019 was another month with very hot and dry weather. The first climatological studies documenting the heat in 2019 concern the Iberian Peninsula (Sousa et al., 2019). Sulikowska and Wypych (2020) have identified Central Europe (and especially Poland) as another hot spot, even hotter than Iberian spot in Europe in June 2019. Both reports concluded that hot June 2019 was an effect of long lasting inflow of southern air. Spatial distribution of heat stress as defined by UTCI, during the heat wave in June 2019 is presented at the Copernicus webpage (https://climate.copernicus.eu/ESOTC/2019/heat-and-cold-stress, ESOTC, 2019). The global ECMWF database of meteorological variables was applied for UTCI calculations and the map presented there shows distribution of particular thermal stress categories. In the period between June 24 and July 2, 2019, several hot spots with very strong heat stress are identified over Western and Central Europe, including western part of Poland. However, till now there are no analyses of biometeorological consequences of this heat wave event in Central Europe.

For people, heat stress conditions can lead to health problems, caused by overloading of thermoregulatory and circulatory systems. This leads in turn to skin eruptions, heat fatigue, heat cramps, heat syncope, heat exhaustion, and heat stroke (Błażejczyk et al. 2018; Köppe et al. 2004). Increased sweat evaporation in hot conditions can cause hazardous health disorders starting from thirst and heat fatigue at 2% decrease of body mass due to water loss through the increase in heart rate and body temperature at dehydration level of 6% up to heat stroke and death at 14% dehydration (Menne and Matthies, 2009).

Dramatic increases in all-cause mortality is commonly observed during heat waves (e.g. Diaz, 2014; Laschewski and Jendritzy, 2002; Green et al. 2016; Tan et al. 2007; Vandentorren et al. 2006). The study conducted by Baccini et al. (2008) in Central European cities estimated that 1°Cincrease at apparent temperature (AT) above 22°Cthreshold leads to 1.3–2.2% increase in mortality. The research of Błażejczyk and McGregor (2008), Bouchama and Knochel (2002) and Kuchcik (2021) suggest that prolonged heat waves without refreshing period during cooler nights lead to the accumulation of heat in the body core.

Air pollution is indicated as additional factor influencing human health. Stagnant atmospheric conditions, especially those associated with heat waves, can lead to concentration of pollutants, and amplify the negative health effects in subjects suffering from circulatory and respiratory diseases (Ren et al. 2006; Saldiva et al. 1995). The results of EuroHeat project (Menne and Matthies, 2009) show that during days with combined effect of heat stress and elevated concentration of tropospheric ozone mortality rates were higher by about 16% in comparison to neutral days. The negative effect of heat stress alone was only 10.5%.

The purpose of this article is to discuss biometeorological and health effects of prolonged European heat wave in June 2019. Attention is paid to their impact on thermo-physiological reactions of human organism as well as modelled and observed heat-related mortality. We will also examine synoptic and climatological background of the long-lasting persistence of high air temperature and heat stress. The research concerns Poland as an area of the biggest thermal anomalies (Sulikowska and Wypych, 2020) and one of the most intensive heat stress spots in Europe (ESOTC, 2019).

## Data and methods

### Climatological background

**Fig. 1:**
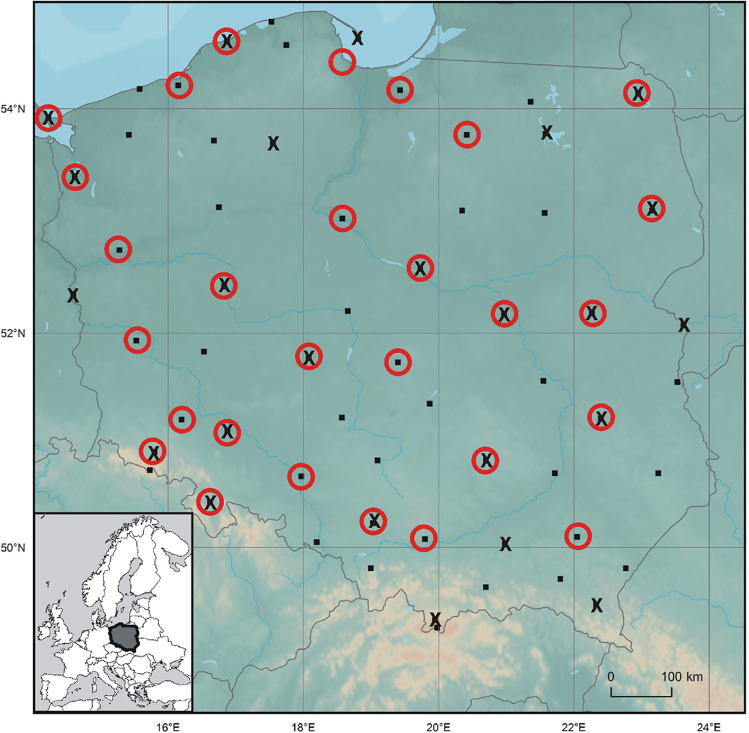
Synoptic stations and cities included in the study; ▪ — synoptic stations, x — stations with multiannual UTCI data, red circles — cities with mortality data. Source: author’s own elaboration

The input for the study are daily and monthly air temperature values in June observed in the span of 1951–2019 at 60 synoptic stations in Poland (Fig. 1). The data is available at the database of the state meteorological service in Poland (IMGW-PIB, https://danepubliczne.imgw.pl/). The magnitude of air temperature anomalies in June 2019 is expressed in absolute (°C) and relative (defined by the magnitude of standard deviation, SD) values of average, maximum and minimum daily temperature. They were used to determine thermal characteristics of June 2019, namely: the number of days with t_max_ > 25, > 30, and > 35^o^C (i.e. hot, very hot, extremely hot days, respectively), number of days with t_min_ > 18 and > 20^o^C (warm and tropical nights, respectively), and the occurrence of heat waves defined as a sequence of 3 days with t_max_ > 30^o^C (Twardosz and Batko, 2012). 30 °C is recognised as air temperature threshold for sudden increase in mortality rates in several European cities (Baccini et al. 2008).

### Synoptic conditions

As reported in previous research, the June 2019 hot event was an effect of air circulation anomalies (Sousa et al., 2019; Sulikowska and Wypych, 2020). The paper therefore presents synoptic background of the hot episode in June. Synoptic maps from the lower and higher levels of atmosphere from www.wetter3.de and www.knmi.nl/home were used for this purpose.

Heat stress phenomenon of July 2019 was compared with the period of 1889–2009 on the basis of the Grosswetterlagen (GWL) calendar of air circulation over Europe. The historical data were taken from Werner and Gerstengarbe (2010) and for 2019 — from https://www.orniwetter.info/wetterlagenkalender/.

### Thermal stress

In human biometeorology, several measures are used for assessing thermal stress. The newest and most complex one is Universal Thermal Climate Index (UTCI) (Jendritzky et al. 2012). In the recent years it was applied in many studies dealing with thermal stress-mortality relationships (e.g. Błażejczyk et al. 2018; Kuchcik, 2021; di Napoli et al. 2018; Urban and Kysely, 2014). The present study is no exception.

In the case of biometeorological characteristics we have applied hourly values of air temperature (ta), vapour pressure (vp), wind speed (v), and total cloud cover (N) for 48 synoptic stations for June 2019. These data were used for the calculation of Universal Thermal Climate Index UTCI. Hourly index values were the basis for calculating average and maximum UTCI values. They were calculated both from 24-h values (UTCIavg, UTCImax) and from midday hours, i.e. 12 UTC (UTCIavg_12, UTCImax_12) which represent daily period of outdoor physical activity in humans. Midday observational term represents as well the hours with the highest air temperature and solar radiation, which are the sources of the highest diurnal level of heat stress. To assess the frequency of various categories of thermal stress, we have analysed a number of days when UTCI represented different intensities of thermal stress, namely: 0.1–9.0 °C (slight cold stress, SCS), 9.1–26.0 °C (no thermal stress, NTS), 26.1–32.0 °C (moderate heat stress, MHS), 32.1–38.0 °C (strong heat stress, SHS), and 38.1–46.0 °C (very strong heat stress, VSHS) (Bröde et al., 2013).

To assess how heat stress in June 2019 differed from bioclimate normals, the data from 24 synoptic stations for the period 1951–2019 were used (Fig. 1). Hourly values of UTCI from midday observational term (12 UTC) were applied to this end, as the UTCI values calculated for 12 UTC represent the time of a day with the highest heat stress. In Central Europe it refers to 14:00 in the summer (CEST) of local time. Deviations of June 2019 from multiannual values of UTCIavg_12 and UTCImax_12 were calculated. Similarly to air temperature, UTCI anomalies are expressed in absolute (°C) and relative (magnitude of standard deviation, SD) values.

The ArcMap 10.5. software package was used to create maps presenting distribution of UTCI characteristics. The Natural Neighbour method, with additional extrapolation based on Kriging, was applied for interpolation.

### Mortality

Thermal conditions of ambient environment influence physiological functioning of the human body and its state of health and wellbeing. Mortality is a common measure of such impact. In the present research we discuss anomalies in June mortality in 2019 in relation to a 10-year period of 2010–2019. Demographic Yearbook of Poland for the years 2011–2020, published by the Demographic Surveys Department of the Statistics Poland, was applied for this purpose (available at: https://stat.gov.pl/obszary-tematyczne/roczniki-statystyczne/roczniki-statystyczne/rocznik-demograficzny-2020,3,14.html).

Relative values of June mortality (TMrel, %) were analysed in relation to mean mortality in 2010–2018. For consecutive years TMrel was calculated as follows:

$$\mathrm{TMrel}=100\cdot\mathrm{ TMx}/\mathrm{TMavg}$$

where: TMx—total number of deaths in particular years in June,

TMavg—total number of deaths in June, average value for 2010–2018.

For 28 cities with population > 100.000 (for which meteorological information from local or nearest station were available) possible mortality attributable to strong heat (SHRM) was also calculated for June 2019 and June 2010–2018. In the calculations, we have used frequency of days with strong and very strong heat stress (SHS + VSHS) in 2019 and in consecutive years from the 2010–2018 period. The following model proposed by Błażejczyk et al. (2018) was adapted for this purpose:

$$\mathrm{SHRM}=2.595\cdot\mathrm{ PopRate }\cdot(\mathrm {SHS}+\mathrm{VSHS})$$

where: PopRate means population rate per 100 000 inhabitants, SHS means number of days with strong heat stress (UTCI of 32.1–38.0ºC) and VSHS means the number of days with very strong heat stress (UTCI > 38.0ºC).

Information concerning population of 28 selected cities were taken from the same source as mortality data. The population of these cities is 8.1 million, which constitute about 21.3% of the total population of Poland. In the studied period (2010–2019), the population of the selected cities was stable and had been changing in the range of 99.7–100.6% year on year, in relation to its average value of examined years.

We have considered both mortality rates possibly attributable to strong heat stress (SHRM) and the mean June SHRM rates in the period 2010–2018 (SHRMrel).

## Heat strain measures

Several physiological indicators were applied to assess heat strain of the human organism, namely: net heat storage (S), heart rate (HR), and sultriness index (HSI). Net heat storage (in W·m^-2^) represents resultant value of heat exchange between human body and the environment. The S value (W⋅m^−2^) is derived from the human heat balance model MENEX_2005 (Błażejczyk and Kunert, 2011).The general equation of the human heat balance has the following form:

$$\mathrm{S}=\mathrm{M}+\mathrm{Q}+\mathrm{E}+\mathrm{C}+\mathrm{Res},$$

where: *M* is metabolic heat production (W⋅m^−2^), *Q* radiation balance of a man consisting of absorbed solar radiation and net long wave radiation exchange (W⋅m^−2^), *E* evaporative heat loss (W⋅m^−2^), *C* heat exchange by convection (W⋅m^−2^), *Res* heat loss by respiration (W⋅m^−2^).

According to Clark et al. (1980), the S of + 15.0 W·m^-2^ was taken as value leading to the risk of overheating. After 3 h of outdoor exposure core temperature of a body may increase by 2.0 °C through accumulation of heat (Smolander, 1987).

Heart rate (in beats per minute) expresses heart load caused by meteorological conditions. HR values depend on metabolism (M, set as 135.0 W·m^-2^ , i.e. typical for person walking 4 km per hour), air temperature (ta, °C) and air vapour pressure (vp, hPa). HR of 90 beats per minute was taken as a warning value as it leads to the overload of circulatory system (Givoni and Goldman, 1973; Michajlik and Ramotowski, 1996). HR is calculated according to Fuller and Brouha (1966) formula:

$$\mathrm{HR}=22.4+0.18\cdot \mathrm{M}+0.25\cdot (5\cdot \mathrm{ta}+2.66\cdot \mathrm{vp})$$

The sultriness index (HSI, in %) was proposed by Belding and Hatch (1955). It indicates thermal-and-humid stress in the human body and is expressed as a ratio of evaporation required for keeping heat equilibrium of an organism to maximal evaporation in actual environmental conditions. HSI is calculated as follows:

$$\mathrm{HSI}=100\cdot \mathrm{Ereq}/\mathrm{Emax}$$

where: Ereq = M + Q + C + Res and Emax = k·v·0.6·(56—vp) (both in W⋅m^−2^), v is wind speed (m⋅s^−1^) at 1.2 m height and k is empirical coefficient equal to 7.0 for clothed and 11.7 for naked person.

HSI > 30% indicates intensive sultriness leading to health risk for the people who have not been acclimatised as well as the elderly, asthma, and hypertension patients (Błażejczyk and Kunert 2011).

To assess the risk of overheating, total time (in hours) when UTCI values exceeded 26 °C (heat stress time, HST) and were below 18 °C (heat recovery time, HRT), was analysed. The limit of HRT is derived from the “thermal comfort zone” concept. According to the *Glossary of Terms for Thermal Physiology* (IUPS 2003), UTCI values between 18 and 26 °C represent “The range of ambient temperatures, associated with specified mean radiant temperature, humidity, and air movement, within which a human in specified clothing expresses indifference to the thermal environment for an indefinite period” (Bröde et al., 2013). Thus, thermal conditions represented by UTCI < 18 °C accelerate elimination of additional heat from an organism and, consequently, lead to its recovery.

Daily totals of hours with S > 15 W·m^-2^ ^-2^, HR > 90 beats per min, HSI > 30% as well as HST (with UTCI > 26 °C) and HRT (with UTCI < 18 °C) were taken as measures of heat strain of human organism.

BioKlima 2.6 software package (https://www.igipz.pan.pl/bioklima.html) was used for the calculation of biometeorological indices. Statistical analyses were made with the use of STATGRAPHIC Centurion software package.

## Air pollution

While searching for the explanation of excess mortality in June 2019, we also considered air pollution indicators. We analysed daily data of PM2.5 and O_3_ concentrations in Poland for June 2019 and for the period of 2010–2019. Mean daily concentrations were taken into consideration for PM2.5 and hourly values for O_3_. The input data from monitoring stations are available at https://powietrze.gios.gov.pl/pjp/archives. For the calculation of spatially averaged PM2.5 values the data from 67 monitoring stations were applied. In the case of O_3,_ data from 105 stations were used.

## Results

### Meteorological conditions

**Fig. 2:**
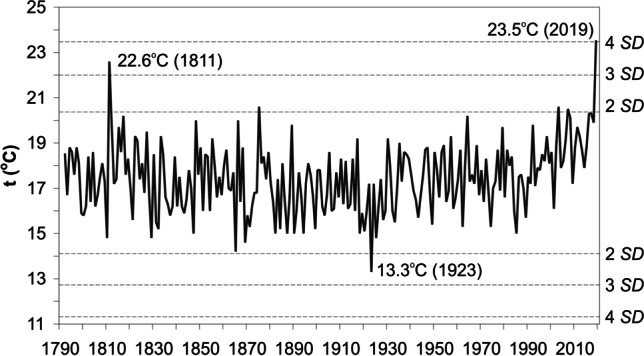
Average air temperature (t) in June for Kraków (1792–2019); multiplied values of standard deviation (SD) are marked with a dashed line. Source: author’s own elaboration

In 2019, after a relatively cool and wet May, warm weather appeared in Central Europe and air temperature in Poland increased very quickly. Average monthly values reached 21–23 °C in June, especially in western Poland (supplementary materials 1). The exceptionally high temperature in June 2019 is apparent in the longest secular series of air temperature in Poland, from Kraków (1792–2019) (Fig. 2). The average temperature there was 23.5 °C (with 4.0 SD) and was higher by almost 1 °C than the temperature of the second in the ranking, June 1811 (22.6 °C, 3.4 SD). The extreme high temperature in June 2019 is evidenced by the magnitude of absolute (up to 6.4 °C in Poznań) and relative (up to 4.9 SD in Piła and Słubice) anomalies compared to the average from 1951–2019.

In June 2019, more than 20 hot and up to 15 very hot days were recorded throughout the country. Warm and tropical nights were also relatively frequent. Two heat waves appeared in June 2019, between 10 and 16 as well as 25 and 27 June (supplementary materials 2).

### Synoptic background

**Fig. 3:**
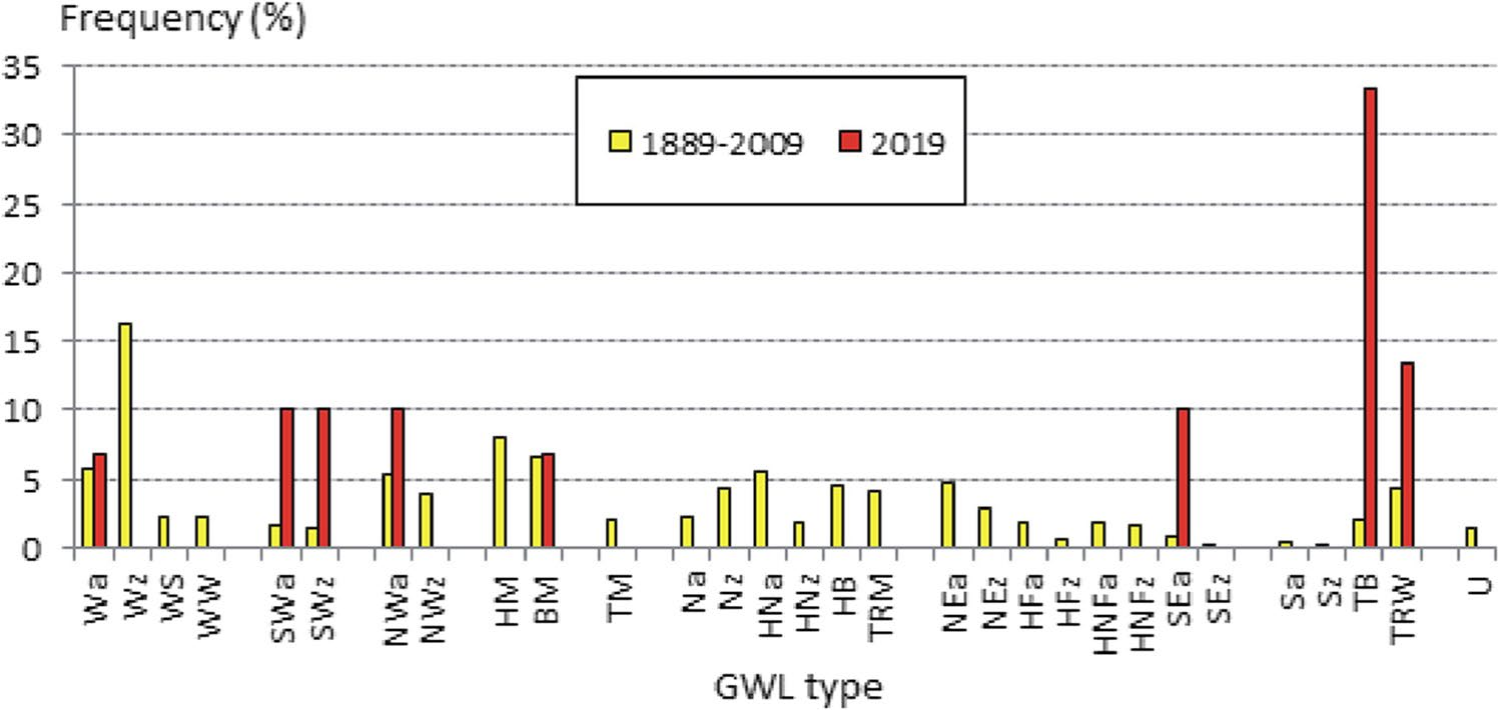
Frequency of GWL types in June 2019 and in the period 1889–2009. Source: author’s own elaboration

A secular data series of GWL (for the period of 1889–2009) shows that two circulation types were most frequent in June in Central Europe, namely: Wz (westerly cyclonic, 16.2% of days) and HM (high over Central Europe, 8.0%). A very atypical circulation system was observed in June 2019. During about 10 days deep atmospheric low (TB type) occurred over British Islands and in 4 days Western European trough (TRW type) was very active. Additionally, during several days SWa and SWz (south-western, anticyclonic and cyclonic circulation) and SEa (south-eastern anticyclonic inflow) types have occurred for 3 days each. All those types cause the warm and even hot air from subtropical zone and from the north of Africa being directed to the Central Europe (Fig. 3).

**Fig. 4:**
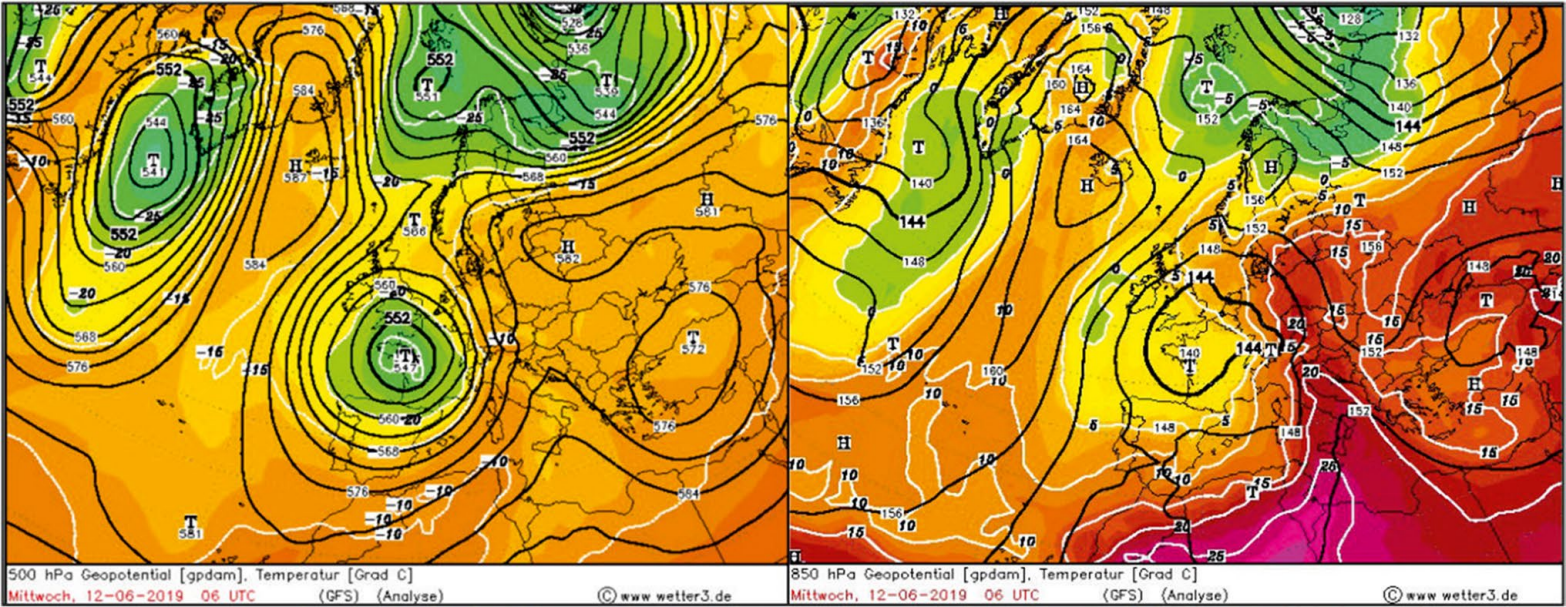
Pressure and temperature field over Europe on 12 June 2019 at 500 (left panel) and 850 (right panel) hPa levels. Source: wetter3.de

The atypical structure in GWL types is confirmed by the analysis of synoptic maps for selected days of June 2019. In the first twenty days the advection of warm air from the southern sector dominated over Central Europe (TB and TRW GWL types). An example of baric centres distribution which led to the inflow of hot tropical air is clearly visible on synoptic maps from June 12, i.e. from the period of the first heat wave (Fig. 4). The advection of very warm air from the south-east and the south also occurred from 25 to 27 June (supplementary materials 3).

## Thermal stress

**Fig. 5:**
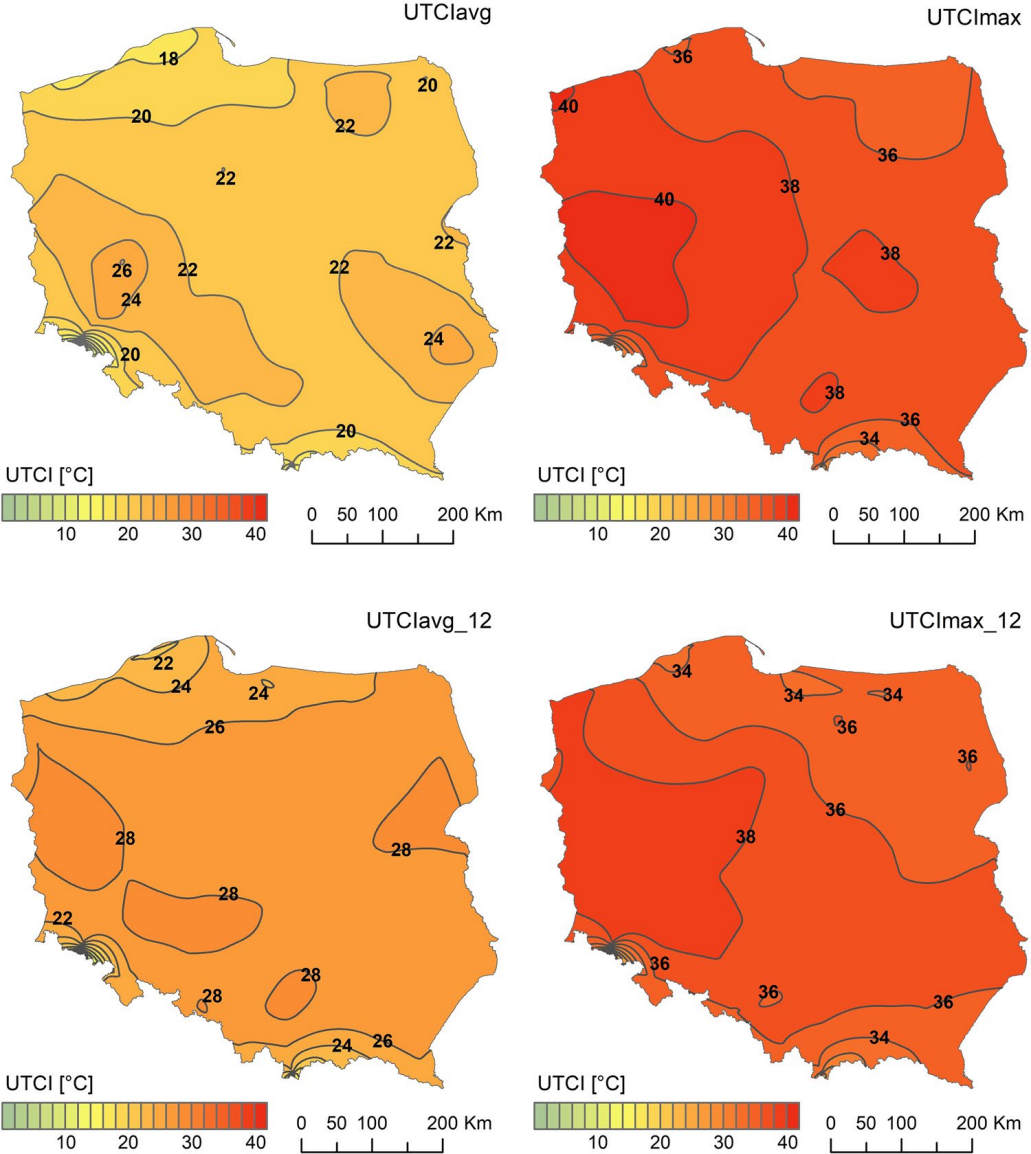
Distribution of specific monthly UTCI values over Poland in June 2019. Source: author’s own elaboration

The mean monthly UTCI values (UTCIavg) varied in the lowland part of Poland from 16.7°C at the Baltic coast to 26.2°C in the west of the country. Maximum monthly index values (UTCImax) fluctuated from about 34°C in NE Poland and in mountains (in the south of country) to approx. 41°C in W and NW Poland. (Fig. 5). The highest mean and maximum UTCI were observed along the Oder river as well as in central and north-western Poland.

Considering thermal stress during midday hours (UTCIavg_12) monthly means were the highest (> 28 °C) mainly along Oder valley and in central part of eastern Poland. The most intensive midday hotspot (UTCImax_12 > 38 °C) was noticed in western Poland. However, relatively mild heat stress (UTCImax_12 < 34 °C) was observed only in few locations in northern and southern Poland (Fig. 5). The comparison of UTCI measures in the years 2010–2019 is discussed in the next chapter.

**Fig. 6:**
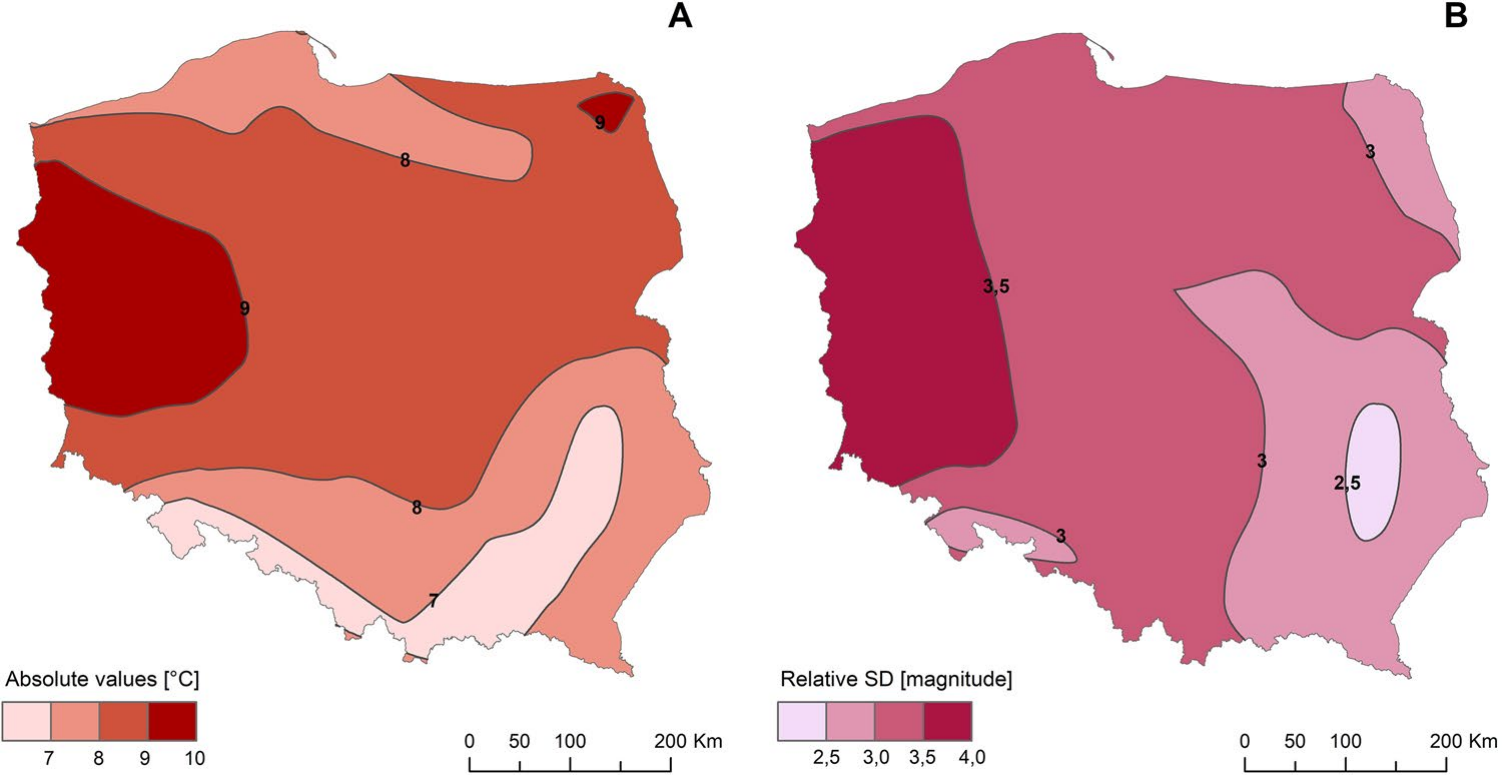
Anomalies of UTCIavg in June 2019 from the period 1951–2019; A — absolute values (°C), B — relative SD (magnitude). Source: author’s own elaboration

Midday UTCI means for June 2019 were significantly higher than in the period of 1951–2018 at all considered stations. Absolute anomalies reach from 6.2 to 6.5 °C in southern and south eastern to > 9 °C in western Poland. At almost all stations relative anomalies are bigger than 3 SD. Only at NE and SE edges they are lower (from 2.3 in Lublin to 2.9 in Suwałki), and at stations along Polish-German border they are even 4 SD (Fig. 6).

**Fig. 7:**
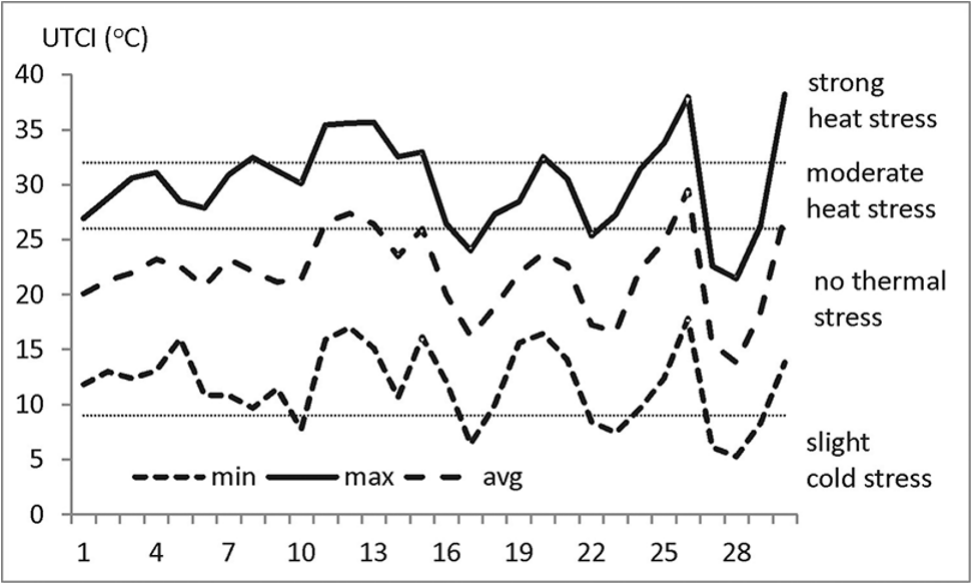
Monthly course of daily 24-h minimum, maximum, and average UTCI values in June 2019 in Warsaw. Source: author’s own elaboration

The analysis of air temperature showed that in June 2019 two heat waves happened and both had great impact on biometeorological conditions. For example, in Warsaw, which represents an area of great intensity of heat wave, on almost all days UTCImax was in the categories of moderate and strong heat stress and on 3 days only UTCImax was lower than 26 °C. Additionally, on 7 days only the night minimum of UTCI was below 9 °C (slight heat stress) which favour night heat recovery (Fig. 7).

### Heat stress mortality

Great and long-lasting heat stress have caused significant increase in total number of deaths in June 2019 incomparison to the period of 2010–2018. Medical records show that TM rates in Poland in June 2019 was almost 10% higher than in the preceding 9 years. One of the causes for the TM increase might be organism overload by strong heat stress. The applied model of SHRM indicates that in selected cities (representing 23% of Poland’s population) the number of deaths attributed to strong heat stress was five times higher in June 2019 than the average for the period of 2010–2018. Thermal stress measures were significantly higher in June 2019 than in the preceding 10 years. UTCIavg_12 value was of 6.8°Cand the number of SHS + VSHS days was 5.6 days higher than in the reference period (Table 1).

**Table 1:** Characteristics of relative mortality (TMrel, SHRMrel) and air pollution (O_3_, PM2.5) in June in particular years in the period 2010–2019

Period	Mortality indicators		Thermal stress measures		Air pollution			
	TMrel (%)	SHRMrel (%)	Mean monthly UTCI (°C)	Number of heat stress days	O_3_ (μg/m^3^)	O_3_ (%)	PM2.5 (μg/m^3^)	PM2.5 (%)
2010	101. 7	115.6	20.0	1.3	61.3	94.9	13.3	108.5
2011	-	45.2	20.5	0.5	67.9	105.2	12.5	102.3
2012	99.5	88.2	17.8	1.0	65.1	100.8	11.9	97.5
2013	98.5	97.4	20.1	1.4	59.9	92.7	13.6	110.9
2014	-	110.9	18.2	0.9	59.4	91.9	11.6	95.2
2015	99.9	89.6	19.4	0.9	66.9	103.6	11.7	95.9
2016	98.6	201.5	22.2	2.5	66.1	102.3	12.1	98.7
2017	99.9	34.8	19.7	0.3	64.8	100.3	11.1	90.8
2018	101.9	116.9	21.6	1.0	69.9	108.2	12.2	100.1
2010–2018 mean	100.0	100.0	20.0	1.1	64.6	100.0	12.2	100.0
2019	109.6	509. 6	26.8	6.7	76.6	118.6	12.3	100.8

Source: author’s own elaboration

Some authors indicated in their studies possible impact of the air pollution, mostly PM2.5 and O_3_, on increased mortality. To verify this we have analysed concentration of these pollutants in Poland in the years 2010–2019. The PM2.5 concentration has not exceeded safe levels either in 2019 or in the preceding 10 years. As for ozone, its mean concentration was approx. 19% higher in 2019 than in the years 2010–2018. However, elevated O_3_ level was also observed in 2011 and 2018 without any visible impact on the TMrel and SHRMrel values (Table 1). The monthly resolution and short period of available data do not allow for deep analysis of the degree to which mortality is affected by air pollution. The results suggest that only O_3_ concentration might have had limited impact on exceeded mortality in June 2019. However, this hypothesis calls for more detailed research in the future.

Apparently, strong heat loads in June 2019 were the main, possible cause of the increased TM and SHRM. Such heat loads were manifested by several physio-climatological indicators. As an example, the calculations of selected indices were made for Warsaw which represents population of about 2 millions.

**Fig. 8:**
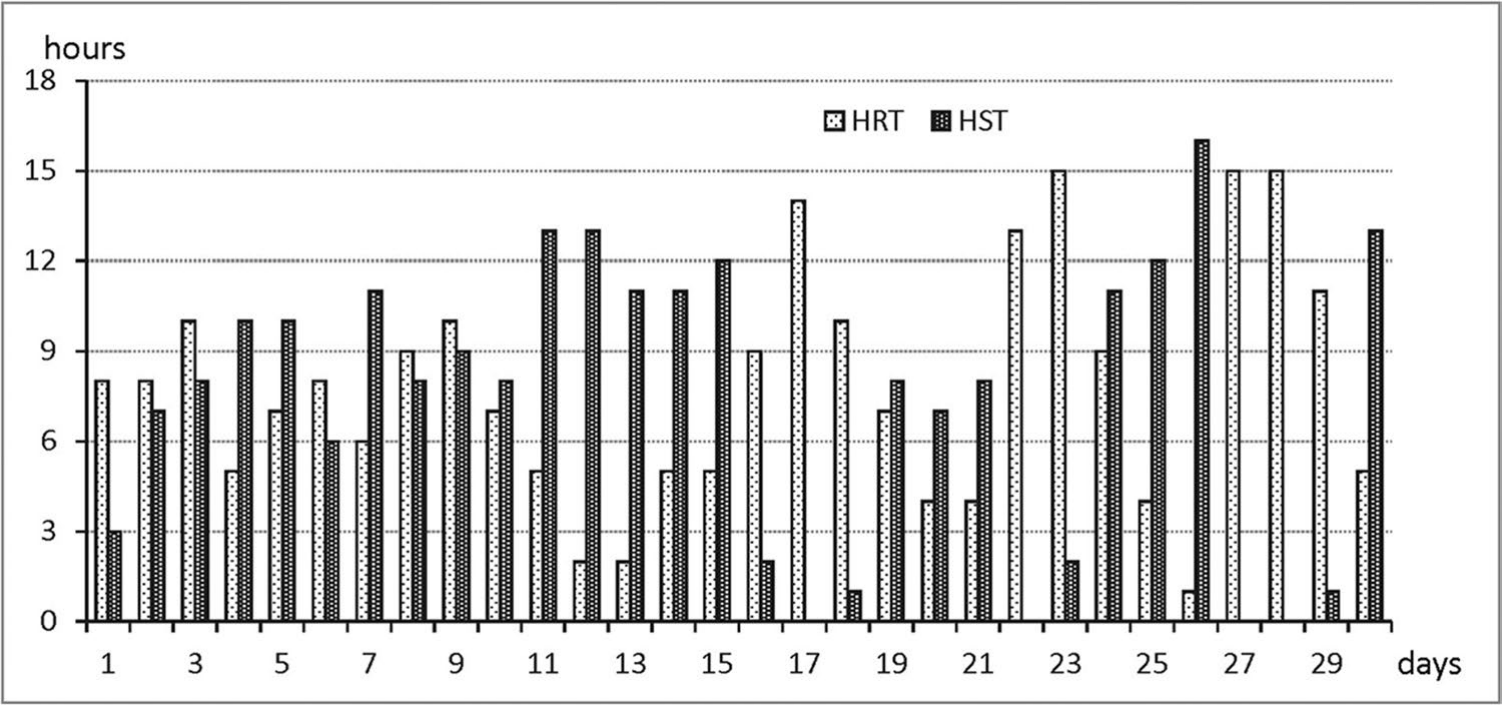
Daily amount of hours with heat stress time (HST) and heat recovery time (HRT) in Warsaw in June 2019. Source: author’s own elaboration

In the previous paragraphs the attention was paid to general synoptic, thermal and heat stress characteristic of June 2019. However, while searching for the explanations of increased mortality in this year, we have analysed several heat strain indicators: HST, HRT, HR and HSI. They provide information of day-by-day 24-h balance of time which can lead to overheating and recovering of a human body. Two periods with elevated heat stress, i.e. 2–15 June (with an extremely hot sub-period of 11–15 June) and 24–26 June were taken into consideration. In the first half of the month thermal stress intensity increased gradually (Fig. 8). The mean daily time of heat stress conditions (HST) was 9.8 h and heat recovery time (HRT) was 6.3 h. In the last 5 days (of the period of 11–15 June) HST was 12 h and HRT— only 3.8 h daily (on 12 and 13 June it was only 2 h daily). At the end of the month the mean HST was 13 h, and mean HRT— 4.7 h (on 26 June 2019 it was only one hour). Such long daily times of heat stress and very short heat recovery times in extremely hot days could lead to heat accumulation and the risk of overheating. This is dangerous especially for elderly people and coronary patients.

**Fig. 9:**
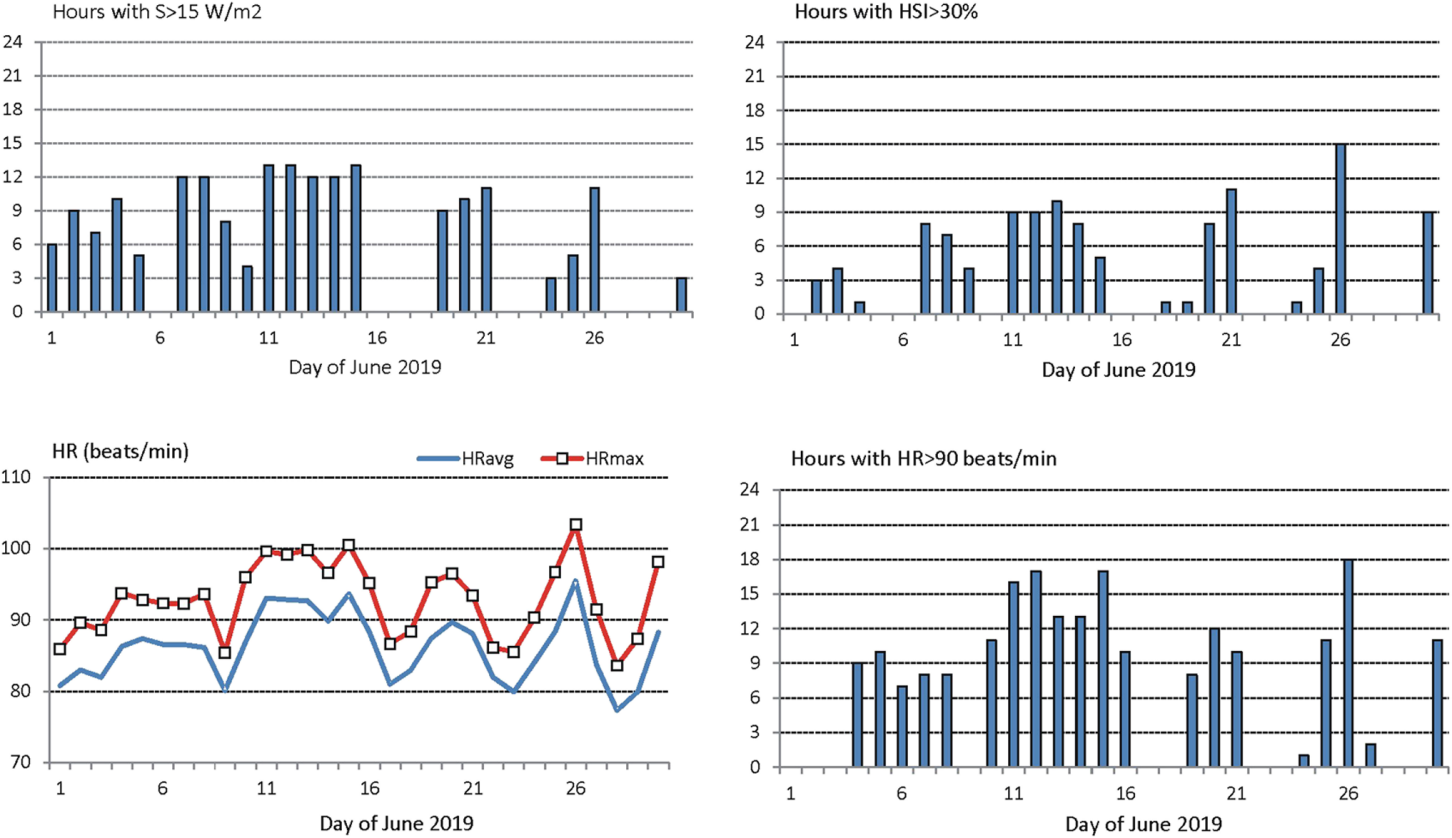
Physiological characteristic of a human organism in June 2019 in Warsaw; S — net heat storage, HSI — sultriness index, HR — heart rate. Source: author’s own elaboration

The results of HST and HRT considerations are confirmed by the analysis of other thermo-physiological indices. Daily amount of hours with net heat storage S > 15 W·m^-^^2^, (which leads to heat accumulation in the body core) in the first half of June lasted up to 12 h daily (as seen on June 7–8 and 11–15 respectively). Daily time balances with great sultriness risk (i.e. with HSI > 30%) were also very long. Between 11 and 15 June these lasted for 4–7 h daily. On June 26 the total daily time with the risk of sultriness was as long as 15 h. Loaded thermal environment led to a great increase of heart rate. Almost throughout the month 24-h means of HR were higher than 80 beats per minute. In most stressed days maximum heart rate was about 100 beats/min. Thermal conditions which led to strong load of heart (HR > 90 beats/min) lasted several hours during particular days. In the periods of 10–16 and 25–26 June they dominated at 10–18 h daily which means that they occurred not only in the daytime but also at night (Fig. 9) This finding confirms very short heat recovery time in those days in June 2019, as showed in Fig. 8.

It seems that all factors presented above indicate great load of human circulatory system which led as a result to increased mortality in population.

## Discussion

The current state of research suggests that the direct cause of the long-term occurrence of extremely high air temperature in the summer months are stationary high pressure systems, forming what is known as blocking situations covering the entire thickness of the troposphere, e.g. in the summers of 2003 (Fink et al., 2004), 2010 (Schneidereit et al., 2012), or in April and May 2018 (Twardosz, 2019). With the normal location of the main baric centres controlling the exchange of air masses, an inflow of hot tropical or polar continental air masses usually occurs, as happened during the above-mentioned extremely warm months. Similar findings are presented by Sulikowska and Wypych (2020), Owczarek et al. (2019), Tomczyk and Owczarek (2019) and by Tomczyk et al. (2020).

The great danger to human health is caused by high air temperatures and intense solar insolation (Köppe et al., 2004; Cheshire, 2016). A marked increase in deaths during hot episodes was reported in many studies (e.g. Vandentorren et al. 2004; Błażejczyk and McGregor, 2008; Nastos and Matzarakis, 2012; Gasparrini et al., 2015). In the studies conducted in recent years, the authors emphasize an increase in mortality and morbidity, especially among the elderly and those with circulatory problems, during days with high UTCI values (Urban and Kyselý, 2014; Kuchcik 2017, 2021; Błażejczyk et al., 2018; di Napoli et al., 2018).

Considering increase in mortality during heat waves one must remember that meteorological information refer to stations located outside the cities. Increase in mortality during heat waves is also affected by the specific urban climate phenomenon, the urban heat island (UHI). The typical pattern for UHI is higher night-time temperature in the city than out of the city centre, which can reduce night heat recovery time. Elderly people (65 +), children, pregnant women, people with chronic somatic and mental disorders or people with disabilities (especially those with limited mobility) have limited capacity to adapt to extreme heat and can be mostly affected by negative influences of UHI (Curriero et al., 2002; Diaz, 2014; Ellis and Nelson, 1978; Flynn et al. 2005; Huynen et al., 2001; d'Ippoliti et al., 2010; Kuchcik, 2017; Naughon et al. 2002; Vandentorren et al. 2006; Yaron and Niermeyer 2004; Ye et al. 2001). Kysely (2004) and Ye et al. (2012) have linked negative health effects of heat waves with what is known as the harvesting effect. It occurs when heat wave lasts for several days. It seems that our results dealing with daily time balance of heat strain indicators also confirm harvesting effect of June 2019 heat stress conditions.

The results of the EuroHeat project concerning mortality during heat waves in 9 European cities shown the rise of mortality with the rising length and intensity of heat. In the Mediterranean cities heat waves led to 21.8% rise of mortality while in Central Europe—12,4% (d’Ippoliti et al., 2010) which is consistent with the present findings. The EuroHeat project also suggested that increased air pollution might be a contributing factor influencing mortality risk. In our research, we were not able to observe direct correlation between air pollution and increased mortality rates. It seems that increased mortality rates were mostly caused by extreme heat stress conditions observed in June 2019.

Heat stress at the individual level leads to inability to thermo-regulate, with the result that body temperature rises and physiological functions begin to break down or fail. Unfit people have limited cardiovascular reserves and low heat tolerance. Heat stress in association with vasodilation and dehydration exacerbates health problems such as cardio-vascular disease (Menne and Matthies, 2009). This is consistent with our findings on the possible impact of the raised heart rate on elevated mortality in June 2019.

Earlier studies show that prolonged heat waves may lead to the accumulation of heat in the body core and, after consecutive hot days without regeneration during cooler nights (as documented in our research), individuals may suffer from thermoregulatory failure (Bouchama and Knochel, 2002; Laschewski and Jendritzky, 2002; Błażejczyk and McGregor, 2008) as reported in present research.

## Conclusions

The long-term persistence of hot weather in June 2019 was determined by the atmospheric circulation prevailing over Central Europe. The days with circulation of air masses from the southern sector clearly dominated.

Hot weather led to elevated UTCI values and high frequency of days with strong heat stress. Severe thermal conditions caused risk of organism overheating as an effect of 10–13 h daily with heat stress and only 1–5 h of night time heat recovery.

It is possible that modelled heat strain measures which indicate possible strong physiological load of cardiovascular system (related to extreme heat stress conditions) might have been the cause of 10% increase in total mortality.

## Supplementary Information

Below is the link to the electronic supplementary material.

Supplementary file1 (DOCX 958 KB)

Supplementary file2 (DOCX 1364 KB)

Supplementary file3 (DOCX 4476 KB)
