# Supplementary material for: Heat strain and mortality effects of prolonged central European heat wave—an example of June 2019 in Poland
Source: Int J Biometeorol. 2021 Oct 26;66(1):149–61. doi: 10.1007/s00484-021-02202-0 (PMC8727406; doi:10.1007/s00484-021-02202-0)
Supplement: Supplementary file 1 — Supplementary file1 (DOCX 958 KB) [file 484_2021_2202_MOESM1_ESM.docx]

Supplementary materials 1

Climatological background of June 2019 hot episode

In 2019, after a relatively cool and wet May, warm weather occurred in Western and Central Europe. In Poland air temperature increased very quickly over all of its territory, with its average monthly value reaching 21-23°C (Fig. S1A). At 4 stations in southern Poland and in Włodawa (eastern Poland) June 2019 was the warmest month in the period 1951-2019. At 28 stations, it was the second or third month in the ranking of the warmest months, which occurred in July 2006 or in August 2015 (Fig. S1B). The extreme high temperature is evidenced by the magnitude of average anomaly compared to the multi-annual average (1951-2019). The highest absolute anomaly (>6°C, 6.4°C in Poznań) was noted in the central-western part of Poland (Fig. S1C). Relative anomalies were not lower than 3 SD and in the majority of the area of Poland they exceeded 4 SD and at western edge of country (Słubice, Piła), the anomalies reached 4.9 SD and they were the biggest monthly relative anomalies that have occurred in Poland since 1951. Slightly smaller anomalies (about 4.7 SD) occurred at stations in central-western Poland (Fig. S1D).


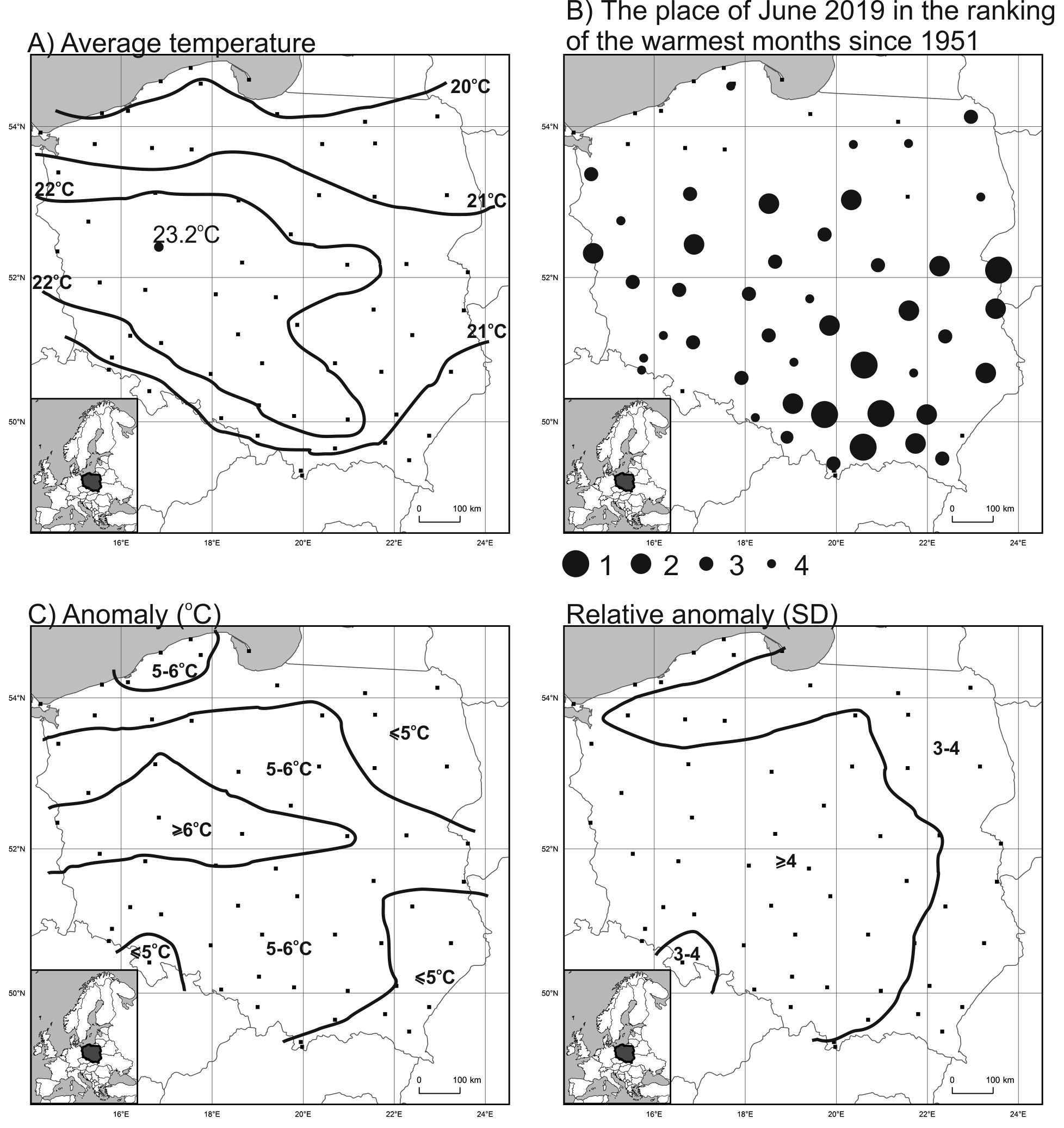


Fig. S1. Characteristics of mean monthly air temperature for June 2019; anomaly values from 1951-2019 period

Source: author’s own elaboration
