# Supplementary material for: Heat strain and mortality effects of prolonged central European heat wave—an example of June 2019 in Poland
Source: Int J Biometeorol. 2021 Oct 26;66(1):149–61. doi: 10.1007/s00484-021-02202-0 (PMC8727406; doi:10.1007/s00484-021-02202-0)
Supplement: Supplementary file 2 — Supplementary file2 (DOCX 1364 KB) [file 484_2021_2202_MOESM2_ESM.docx]

Supplementary materials 2

Magnitude of air temperature anomalies in June 2019

In June 2019 more than 20 hot days were recorded (Fig. S2A). Very hot days occurred all over Poland with different frequency (from 1 to 15 days). The fewest (<5) were along the Gulf of Gdańsk and the most frequent (over 10 days) in central-western Poland (Fig. S2B). In western Poland, there were also 1-2 extremely hot days. Warm and tropical nights were relatively frequent and distributed all over Poland (Fig. S2C, S2D). Two heatwaves appeared in June. The first of them, with different lengths at individual stations (3-7 days), occurred between 10 and 16 June, covering most of Poland (Fig. S2E). The second 3-day heat wave, with record temperature of up to 38°C, between June 25 and 27 covered Central and Western Poland (Fig. S2F).


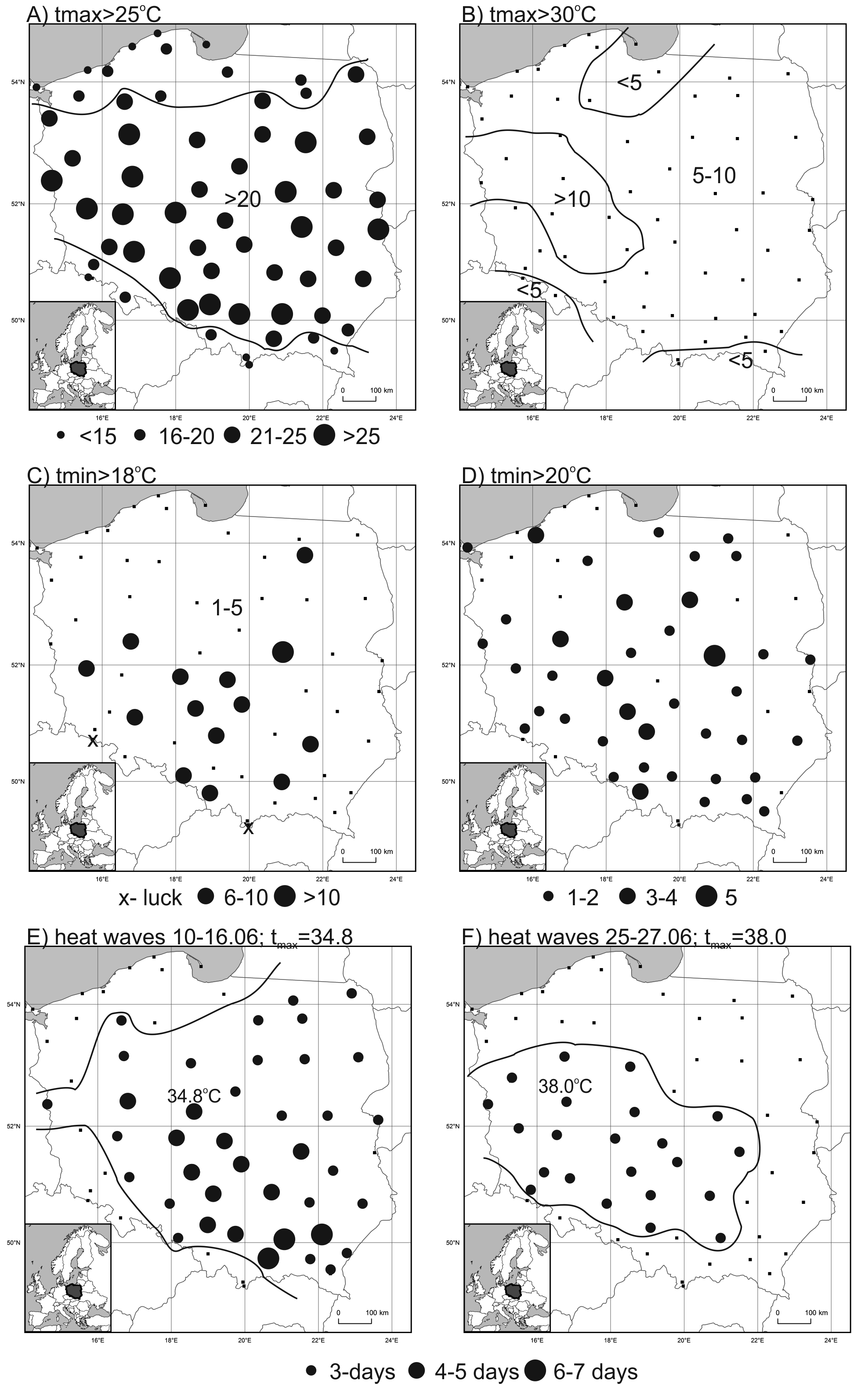


Fig. S2. Number of hot (tmax>25°C, A) and very hot (tmax>30°C, B), warm (tmin>18°C, C) and tropical (tmin>20°C, D) as well as length of heatwaves (E, F) in June 2019

Source: author’s own elaboration
