# Supplementary material for: Heat strain and mortality effects of prolonged central European heat wave—an example of June 2019 in Poland
Source: Int J Biometeorol. 2021 Oct 26;66(1):149–61. doi: 10.1007/s00484-021-02202-0 (PMC8727406; doi:10.1007/s00484-021-02202-0)
Supplement: Supplementary file 3 — Supplementary file3 (DOCX 4476 KB) [file 484_2021_2202_MOESM3_ESM.docx]

Supplementary materials 3

Synoptic background of June 2019 hot episode

In the first twenty days of June 2019 advection of warm air masses from the southern sector dominated over Central Europe. In the Eastern Europe there was a well-developed and extensive high-pressure system, and over western Europe and the Atlantic a multi-centre low pressure system. Such distribution of baric centres led to intensive inflow of hot tropical air mass from the south, which is clearly visible at 850 hPa level, where the temperature was about 20°C as shown on synoptic maps from June 12, i.e. from the period of the first heatwave (Fig. 3). At many meteorological stations, the air temperature was approximately 34°C. The hottest days occurred during the 3-day heat wave from 25 to 27 June which was caused by the advection of very warm air from the south-east and the south. The lack of cloud cover and very low water vapour content favoured the intense heating of air (up to 38°C). The air temperature at 850 hPa has raised from 15°C on 25 June, and on June 26 it exceeded 22°C (Fig. S3A). On those days, in the central troposphere (500 hPa), the area of Central Europe was influenced by the ridge of high pressure from northern Africa. Low pressure systems prevailed in the east and west of Europe. In the lower troposphere, the high pressure system was moving from the Baltic Sea to eastern Europe. From the afternoon of June 26, Poland began to come under the influence of the trough associated with the low pressure from Scandinavia which brought gradual and intensive heating (Fig. S3B).


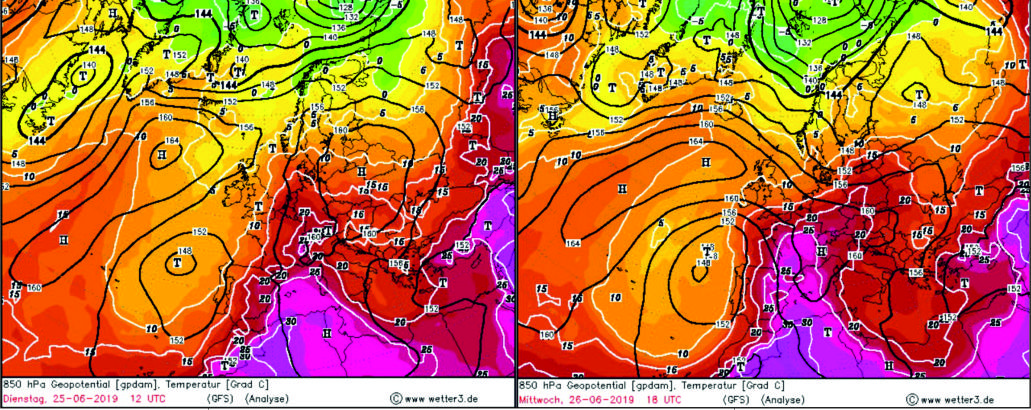


Fig. S3A. Pressure and temperature field over Europe on 25 (left panel) and 26 June 2019 (right panel) at 850 hPa

Source: wetter3.de


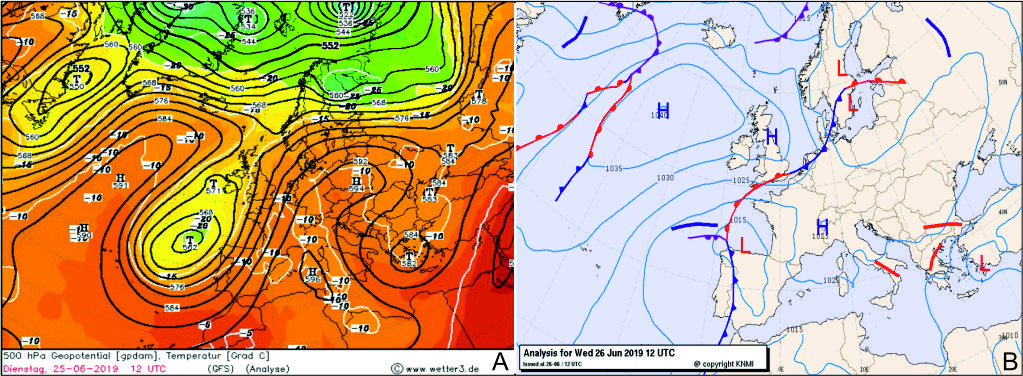


Fig. S3B. Pressure and temperature field over Europe on 25 June 2019 at 500 hPa (left panel) and synoptic situation at 12:00 UTC (right panel) on 26 June 2019

Source: wetter3.de
